# LAO-NCS: Laser Assisted Spin Torque Nano Oscillator-Based Neuromorphic Computing System

**DOI:** 10.3389/fnins.2019.01429

**Published:** 2020-01-22

**Authors:** Hooman Farkhani, Tim Böhnert, Mohammad Tarequzzaman, José Diogo Costa, Alex Jenkins, Ricardo Ferreira, Jens Kargaard Madsen, Farshad Moradi

**Affiliations:** ^1^Integrated Circuits and Electronics Laboratory, Department of Engineering, Aarhus University, Aarhus, Denmark; ^2^International Iberian Nanotechnology Laboratory, Braga, Portugal

**Keywords:** neuromorphic computing system, laser, power efficient, COMSOL multiphysics, spin torque nano-oscillators

## Abstract

Dealing with big data, especially the videos and images, is the biggest challenge of existing Von-Neumann machines while the human brain, benefiting from its massive parallel structure, is capable of processing the images and videos in a fraction of second. The most promising solution, which has been recently researched widely, is brain-inspired computers, so-called neuromorphic computing systems (NCS). The NCS overcomes the limitation of the word-at-a-time thinking of conventional computers benefiting from massive parallelism for data processing, similar to the brain. Recently, spintronic-based NCSs have shown the potential of implementation of low-power high-density NCSs, where neurons are implemented using magnetic tunnel junctions (MTJs) or spin torque nano-oscillators (STNOs) and memristors are used to mimic synaptic functionality. Although using STNOs as neuron requires lower energy in comparison to the MTJs, still there is a huge gap between the power consumption of spintronic-based NCSs and the brain due to high bias current needed for starting the oscillation with a detectable output power. In this manuscript, we propose a spintronic-based NCS (196 × 10) proof-of-concept where the power consumption of the NCS is reduced by assisting the STNO oscillation through a microwatt nanosecond laser pulse. The experimental results show the power consumption of the STNOs in the designed NCS is reduced by 55.3% by heating up the STNOs to 100°C. Moreover, the average power consumption of spintronic layer (STNOs and memristor array) is decreased by 54.9% at 100°C compared with room temperature. The total power consumption of the proposed laser assisted STNO-based NCS (LAO-NCS) at 100°C is improved by 40% in comparison to a typical STNO-based NCS at room temperature. Finally, the energy consumption of the LAO-NCA at 100°C is expected to reduce by 86% compared with a typical STNO-based NCS at the room temperature.

## Introduction

The grand challenge of exascale computing, 10^18^ operations/second, calls for a dramatic change in hardware of the current petascale supercomputers. A paradigm shift is needed to tackle the issue of processing the explosively growing Big Data from different sources, which are mostly images and videos as the most time and power-consuming task for the existing Von-Neumann computing machines (VNCs). Filling the gap between the performance of the current computing systems and the brain requires development of a computing system with similar features as the brain; brain-inspired computing systems, so-called neuromorphic computing systems (NCSs). Such systems overcome the limitation of the word-at-a-time thinking of the VNCs by massive parallel data processing similar to the brain (U.S. Department of energy, 2015; DeepMind, 2018; Hbp, 2018; Ibm, 2018; SpiNNaker, 2018). An NCS includes many parallel processors (neurons) communicating using simple messages (spikes) through programmable memory units (synapses). Although significant progress has been made in the CMOS implementation of NCSs, there are some fundamental limits to the simultaneous improvement of area and power in CMOS-based NCS (Fong et al., 2016). Such limits have driven a significant effort to investigate beyond-CMOS NCSs. The spin-based devices integrated with electronics (i.e., spintronics) have opened a door for designers to implement low-power high-density NCSs. In spintronic-based NCSs, magnetic switching in magnetic tunnel junction (MTJ) (Fong et al., 2016) or magnetic oscillation in spin-torque nano-oscillator (STNO) (Yogendra et al., 2015, 2016; Kurenkov et al., 2019) is used to mimic neuron firing. While using oscillation of magnetic moment decreases the power consumption by an order of magnitude compared with the magnetic moment switching [critical current: ∼10^6^ Acm^–2^(Costa et al., 2017) vs. ∼10^–7^Acm^–2^ (Fukami et al., 2016)], still there is a huge gap between spintronic-based NCSs and the brain in terms of power consumption and speed. This is due to the fact that the traditional way of oscillating the magnetic moment through the bias current consumes high power and it is done at low speeds. Hence, there is a crucial need for eliminating or decreasing the bias current in spintronic-based NCSs.

Magnetic tunnel junctions and STNOs can be used to perform Bayesian computation in networks inspired by cortical microcircuits of pyramidal stochastic neurons. This type of neurons spikes stochastically, observed in the cortex (Sengupta et al., 2016). The membrane voltage of a cell can change from the rest potential to oscillatory mode as a result of bifurcation (Bose, 2014). This is very similar to what happen inside STNOs, where the magnetization of FL starts to oscillate by increasing the current passing through the STNO to the currents higher than critical current (Hopf bifurcation). On the other hand, STNOs can show different precession modes based on their bias current (out-of-plane precession and in-plane precession with small or large angle), which are as the result of different bifurcation types, e.g., Hopf bifurcation causes in plane precession and heteroclinic bifurcation leads to out-of-plane precession (Nakada and Miura, 2016). However, in this work, the STNOs with in-plane precession have been used and in order to mimic neuron firing the transition from the magnetization resting state (non-oscillating) to the magnetization oscillation is utilized. It should be noted that the STNOs cannot be used to mimic all bifurcations, for example STNOs unable to mimic SNIC (saddle node on an invariant circle) bifurcation where the f-I curve is continuous (Bose, 2014). In neural networks inspired by biological behavior, the activation function represents the rate of action potential firing in the cell (Hodgkin and Huxley, 1952). In this manuscript, STNOs are used to implement the binary activation function, which is widely used to implement the linear perceptrons in neural networks. The weakness of this type of activation function is that the number of neurons needed for achieving a certain amount of accuracy increases. However, the main goal of this manuscript is to investigate the proof-of-the-concept of improving the performance of the STNO-based systems by elevating the temperature of the STNOs using laser illumination. The STNO-based NCS is used as an application to explore the effectiveness of the proposed idea.

In this manuscript, for the first time to our knowledge, we propose to design a laser-assisted STNO-based NCS (LAO-NCS) to improve power consumption of the state-of-the-art NCSs by at least 40%; narrowing the gap of power efficiency between the Brain and the current NCSs.

### Spin Torque Nano-Oscillators Basics

**FIGURE 1:**
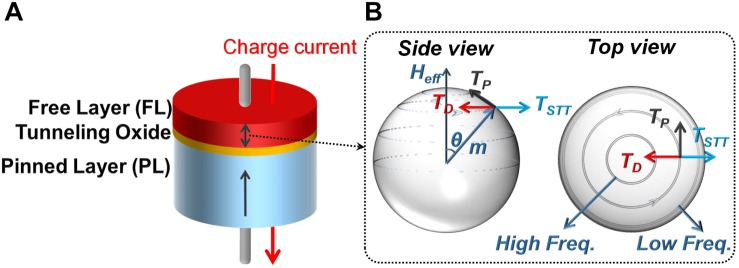
**(A)** The schematic view of a MTJ as spin torque nano-oscillators (STNO) and **(B)** the magnetization direction of MTJ free layer (FL) and torques acting on it.

The schematic of an STNO is shown in Figure 1A, which consists of a Pinned Layer (PL) with fixed magnetization and a free layer (FL) with changeable magnetization direction separated by a tunneling oxide layer e.g., MgO or Al_2_O_3_. Figure 1B shows the magnetization direction of the free layer (m) and different torques acting on it (Yogendra et al., 2015). T_P_ describes the precession torque that leads to the oscillation of m. T_D_ is the damping torque that aligns m with H_eff_ and T_STT_ is the spin-transfer torque caused by a bias current (Yogendra et al., 2016). The interaction of T_STT_ and T_D_ determines the oscillatory orbit of m. As T_STT_ increases, m will be placed in an orbit farther than H_eff_, which will lead to a lower frequency of oscillation of m as shown in Figure 1B (Csaba and Porod, 2013). It is shown experimentally and through simulation that the frequency of the STNO can be locked to the frequency of an RF current passing through it (Rippard et al., 2005, 2013) or an external oscillating RF field (Slavin et al., 2010). Moreover, the frequency of two STNOs can lock if they are close to each other (Kaka et al., 2005). In STNO-based NCSs, the frequency locking of the STNO and comparing its output power with a threshold power are two mechanisms used to implement neuron firing. However, in all cases, a very high DC current (i.e., bias current) is needed flowing through the STNO to generate the required torque (i.e., T_STT_).

### Effect of Raising Temperature on Spin Torque Nano-Oscillators

The dynamic behavior of the FL magnetic moment is modeled using Landau-Lifshitz-Gilbert-Slonczewski (LLGS) equation as follows (Sengupta et al., 2016):

(1) $$\frac{\left(1+\alpha^{2}\right)}{\left|\gamma \right|}\,\frac{\partial \hat{m}}{\partial \tau }=-\hat{m}\times {\vec{H}}_{E\,F\,F}-\alpha \,\hat{m}\times \hat{m}\times {\vec{H}}_{E\,F\,F}+\frac{1}{q\,N_{s}}\,\left(\hat{m}\times I_{s}\times \hat{m}\right)$$

where, α, and $\hat{m}$ are the gyromagnetic ratio, Gilbert damping factor and magnetization of the FL, respectively. ${\vec{H}}_{E\,F\,F}$ is the effective magnetic field acting on FL described by ${\vec{H}}_{E\,F\,F}={\vec{H}}_{U\,A}+{\vec{H}}_{e\,x}+{\vec{H}}_{T\,F}$, where ${\vec{H}}_{U\,A}$, ${\vec{H}}_{e\,x}$, and ${\vec{H}}_{T\,F}$ are uniaxial anisotropy field, external magnetic field and thermal fluctuations field, respectively. $N_{s}=\frac{M_{S}\,V}{\mu_{B}}$ is the number of spins in the FL of volume V (*M*_*S*_ is the saturation magnetization and μ_*B*_ is Bohr magneton) and *I*_*s*_ is the input spin current. The first term in (1) represents the precession torque (T_*P*_) that makes $\hat{m}$ precess around the easy axis. The second term is the damping term (T_*D*_) that tries to align $\hat{m}$ with easy axis. The third term represents the transverse component of spin current being absorbed by $\hat{m}$ (T_*STT*_). In the absence of third term (no current passing through STNO), and in the equilibrium, $\hat{m}$ is aligned with easy axis. By increasing the current, the third term starts to increase, and m starts to oscillate around the easy axis. Higher currents will make the $\hat{m}$ to be placed on orbit farther than easy axis (i.e., higher output power). Increasing the temperature affects the dynamic behavior of the FL through decreasing the saturation magnetization (M_*S*_) of it, decreasing the resistance of the STNO and increasing the dispersion of the initial deviation of the magnetic moment from easy axis due to higher thermal fluctuations.

#### Saturation Magnetization

It is shown theoretically (Ashcroft and Mermin, 1976) and experimentally (Alzate et al., 2014) that the dependency of M_*S*_ can be well described by Bloch’s law as follows:

(2) $$M_{S}\,\left(T\right)=M_{S}\,\left(0\right)\,\left(1-{\left(T/T^{{}^{*}}\right)}^{\frac{3}{2}}\right)$$

where *T* is the absolute temperature in Kelvin and *M*_*S*_(0) is the saturation magnetization at 0K, and *T*^∗^ is a fitting factor. Equation (2) shows that increasing the temperature decreases the M_*S*_(T). This will lead to a degradation of the uniaxial anisotropy field, which decreases the minimum current required for the FL magnetic oscillation.

#### Resistance

Two tunneling mechanisms contribute to the STNO resistance including electron spin-polarized direct elastic tunneling and spin independent tunneling. The total conductance of the STNO can be described as (Teixeira et al., 2010).

(3) $$G\,\left(\theta \right)=G_{T}\,\left[1+P_{1}\,P_{2}\,\cos\theta \right]+G_{S\,T}$$

where θ is the angle between the magnetization of the FL and the PL. P_1_ and P_2_ are the effective tunneling spin polarization of the magnetic layers. G_*T*_ is the pre-factor for direct elastic tunneling. All these parameters are temperature-dependent. Elevating the temperature will increase G_*T*_ and reduces P_1_ and P_2_ (Teixeira et al., 2010). As a result, R_*P*_ is almost independent of temperature while R_*AP*_ reduces approximately linearly with temperature. This has been experimentally shown in Teixeira et al., 2010, Takeuchi et al., 2015, and Hu et al., 2016.

#### Thermal Fluctuations

The effect of temperature on random fluctuating field can be modeled by ${\vec{H}}_{T\,F}$ while its *x*, *y*, and *z* components have uncorrelated Gaussian distribution with zero mean and $\sqrt{\left(2\,\alpha \,k_{B}\,T\right)\,/\,\left(\gamma \,M_{S}\,V\,△\,t\right)}$ standard deviation (Brown, 1963; Sankey et al., 2005; Yogendra et al., 2017). α, k_*B*_, γ, V, and Δt are the Gilbert damping parameter, the Boltzmann’s constant, the gyromagnetic ratio, the volume of the FL and the integration time step. Elevating the temperature will increase the dispersion of ${\vec{H}}_{T\,F}$, which leads to an easier oscillation of the FL magnetic moment. In order to explore the mentioned effects on oscillation behavior of the STNO at elevated temperatures, different characteristics of the STNO (e.g., resistance, TMR, and output power of the oscillation) have been measured at different temperatures from 27°C up to 100°C in section “Memristor Behavior at Elevated Temperatures.”

### Memristor Behavior at Elevated Temperatures

Tantalum-oxide (TaO_*x*_) memristors are one of the best candidate in memory and NCS applications due to their unique characteristics such as CMOS compatibility (Diokh et al., 2013), low power operation (Strachan et al., 2011), high endurance (Lee et al., 2011), and long retention of states (Ninomiya et al., 2013). The conduction mechanism of the TaO_*x*_ memristors can be modeled by two parallel conduction mechanisms including hopping conduction and Schottky thermionic emission (Graves et al., 2017) as follows:

(4) $$I_{t\,o\,t\,a\,l}=\underset{I_{H\,o\,p}}{\underbrace{2\,e\,l\,v_{p\,h}\,N\,k_{B}\,T\,e^{\left(-\frac{W}{k_{B}\,T}\right)}\,e^{\left(-\frac{2\,l}{\zeta }\right)}\,s\,i\,n\,h\,\left(\frac{q\,l\,F}{k_{B}\,T}\right)}}+\underset{I_{S\,c\,h}}{\underbrace{A\,T^{2}\,e^{\left(-\frac{\phi_{B\,0}-\beta \,\sqrt{F}}{k_{B}\,T}\right)}}}$$

where, *k*_*B*_ is the Boltzmann constant, *I* is the hopping distance, *W* is the hopping energy, ζ is the wave function localization, *F* is the applied field (converts from V), *T* is the temperature, *v*_*ph*_ is the vibrational phonon frequency, *A* is the reduced effective Richardson constant multiplied by active device area, *ϕBo* is the barrier height, and β is the barrier lowering factor. *N* is proportional to the density of electrons in the conduction path multiplied by the relevant conducting area. Based on this model, which is well fitted with experimental results, the temperature dependence of TaO_*x*_ memristor resistance can be divided into two regions called cold and hot regions (Graves et al., 2017). In the cold region (*T* ≤ 350K), the state-dependent hopping conduction is dominant and the resistance of memristor is almost temperature insensitive. In the hot region, however, the Schottky emission of electrons determines the hot current, and the memristor’s resistance decreases with raising the temperature, rapidly. Note that, the amount of resistance change of memristor in hot region depends on the memristor’s initial resistance.

### Proposed Laser Assisted Neuromorphic Computing System

**FIGURE 2:**
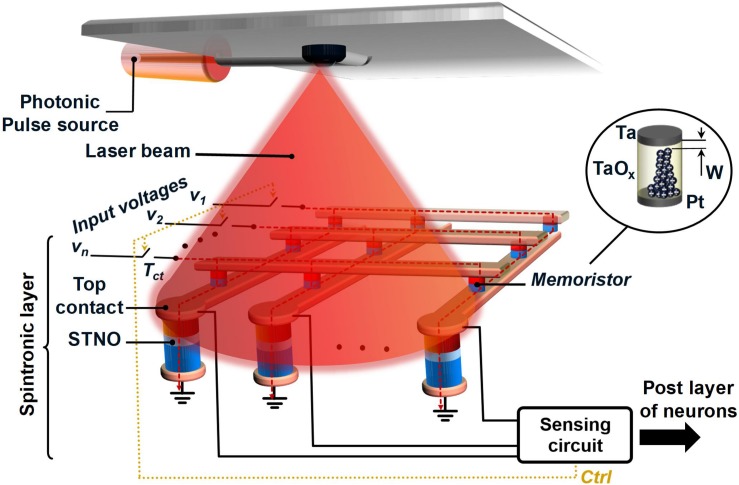
The schematic view of the novel LAO-NCS. The STNO and memristor act as neuron and synapse, respectively. The STNOs will be heated to 100°C by illuminating a laser pulse.

Our novel envisioned LAO-NCS is shown in Figure 2, which is a crossbar array of programmable TaO_*x*_ memristors as synapses and the STNOs as neurons assisted thermally by a narrow laser-pulse. Considering the fact that in many applications, size of the memristor array is much larger than the area of the STNO-based neurons, there is no significant area improvement in stacking the memristor array on top of the STNOs. Hence, the memristor array and the STNOs are supposed to be next to each other in the proposed LAO-NCS. Moreover, this structure allows direct laser illumination on the STNOs’ top contacts. The resistance of the memristors can be tuned using an electric signal flowing through them. The NCS operation starts with a calibration phase in which the temperature of the STNOs will be elevated to 100°C and stabilized. Then, the NCS is ready for operation and the processing phase will start. The processing phase can be divided into two steps including stimulation and recovery, which will be repeated in sequence. In the stimulation step, the crossbar array sums the weighted input currents passing them to the STNOs, which are already set in AP-state (the magnetization direction of the FL and the PL are anti-parallel). In case, the weighted input currents are sufficiently large, the FL magnetic moment of the STNO starts to oscillate that will be detected by a sensing circuit immediately, and translated to neuron firing. The sensing circuit should use track and terminate method (Farkhani et al., 2017, 2018; Torrejon et al., 2017) in order to minimize the energy consumption of the NCS. Immediately after detecting the STNO oscillation, the recovery step begins. In the recovery step, the input corresponding to the fired neuron will be activated in the post-synaptic neuronal layer. Note that one of the advantages of using oscillation instead of magnetic moment switching is that there is no need for switching back the FL magnetization. Hence, the recovery step can be done in a very short time (∼600 ps) compared with the magnetic moment switching (∼2 ns) without extra energy consumption for switching back the magnetic moment. In our approach, the energy consumption needed for starting the STNO oscillation will be lowered significantly by increasing the temperature of the STNO using a nanosecond laser pulse. In fact, increasing the temperature of the STNO will decrease its energy barrier, which leads to a lower bias current needed for starting the oscillation in the STNO. On the other hand, in case, the temperature of TaO_*x*_ memristor array increases to temperatures above 350K due to heat propagation, the resistance of memristors will decrease, as discussed in previous section. However, it seems unlikely that the memristor array temperature reaches above 350K due to limited laser power. Moreover, in order to keep the memristor temperature below 350K, a thermal insulator layer can be placed between the memristor array and the STNOs. Considering the fact that the STNO current passes through the memristor array, the total power consumption of the memristor array will be reduced, significantly. As a result, the power consumption of the LAO-NCS decreases compared with typical spintronic-based NCSs. Considering the fact that the control transistors (T_*ct*_) act as switches, heating them up has no significant impact on the overall performance of the LAO-NCS.

### Interaction Between Laser and the Spin Torque Nano-Oscillators

The on-chip laser can be achieved through vertical cavity surface emitting laser (VCSEL) (Chen et al., 2014; Zhou et al., 2015; Kozlov and Carusone, 2016). VCSEL’s unique specification is that, in contrast to the conventional edge-emitting semiconductor lasers, its laser beam is emitted perpendicular to its surface, which makes it a proper candidate for on-chip laser applications including the LAO-NCS. The output power of VCSEL can be tuned through changing the supply voltage of its driver (Kozlov and Carusone, 2016). Hence, in order to control the output power of the laser, a CMOS interface circuit is designed, which is described below.

### CMOS Interfacing Circuit

**FIGURE 3:**
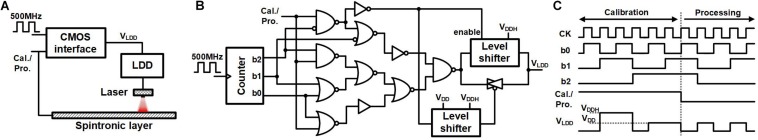
**(A)** The block diagram of the proposed LAS-NCS including CMOS interfacing block, VCSEL array, LDD, and the spintronic layer. **(B)** CMOS interfacing circuit design. **(C)** Timing diagram of the CMOS interfacing block.

Figure 3A shows the block diagram of the proposed LAO-NCS. The spintronic layer includes a memristors array, STNOs, T_*c*_, and a sensing circuit. The CMOS interface circuit adjusts the output laser power by manipulating the supply voltage of the laser diode driver (LDD). In this way, the CMOS interfacing block can control the STNO temperature in the spintronic layer. Figures 3B,C show the circuit design of the CMOS interfacing block and its timing diagram, respectively. As mentioned before, the LAO-NCS operating time can be divided to *calibration* and *processing* phases. In the calibration phase, the temperature of the STNO is increased from 27°C to 100°C (first laser pulse with high power), and stabilized at this temperature (second laser pulse with low power). In the processing phase, the STNO temperature will be kept at 100°C with a sequence of low power laser illuminations as shown in Figure 3C. The operation of the CMOS interfacing circuit is as follows. The counter is clocked with a 500 MHz clock and generates the b0, b1, and b2 signals. Then, the logic circuit generates the V_*LDD*_ signal from the output of the counter. During the first pulse of V_*LDD*_, the level shifter is enabled by a logic circuit and the voltage of V_*LDD*_ will be set at V_*DDH*_ that leads to a high output power laser pulse. During the next pulses, the transmission gate is enabled and the level shifter is disabled by the logic circuit. Hence, the voltage of the V_*LDD*_ node is at V_*DD*_ and the laser output power will be lower.

### Neurons’ Readout Approach

**FIGURE 4:**
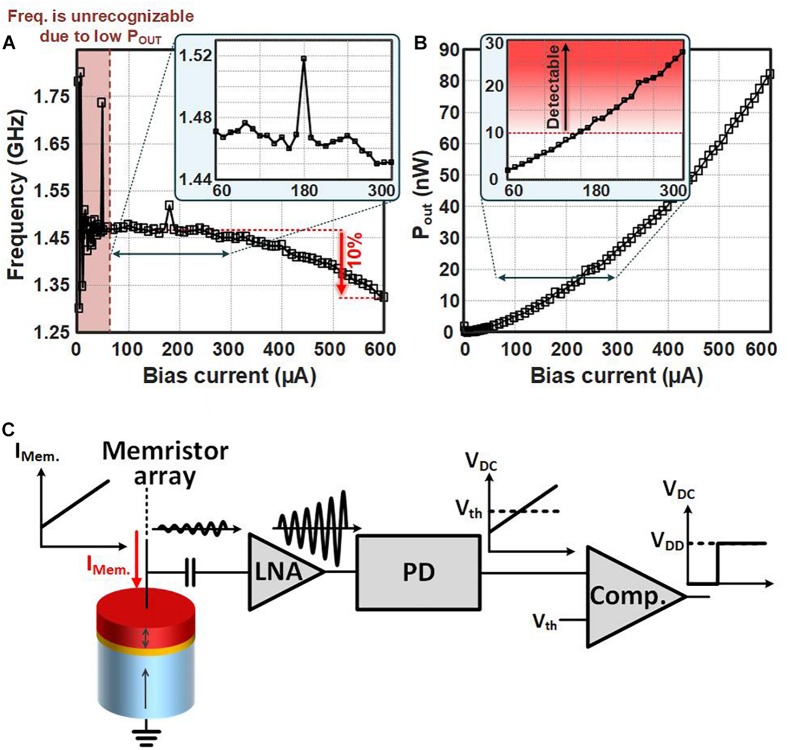
The measured **(A)** frequency and **(B)** output power of the STNO versus different bias current from 0 to 600 μA. The frequency of oscillation is unrecognizable from noise at I_Bias_<60 μA due to low output power of oscillation. The maximum frequency change is 10% @ I_Bias_=600 μA. Output powers higher than 10 nW are detectable by sensing circuits. **(C)** The schematic view of the neuron firing detection approach.

The sensing circuit is to sense the magnetization oscillation of the STNOs (neurons) in order to find the fired neuron(s) and activate the corresponding input(s) in the post-synaptic neuronal layer. This can be done either by sensing the frequency or the output power of the oscillating signal across the STNOs, and comparing it with a threshold frequency or a threshold output power. Figures 4A,B shows the measured frequency and the output power of our STNO samples in response to different bias currents. At bias currents lower than 60 μA, the output power of oscillation is very low. As a result, the frequency of oscillation is not detectable. By increasing the bias current, the frequency of oscillation decreases. However, the frequency reduction rate is slow (just 10% frequency reduction at 600 μA). Hence, it is difficult to detect the fired neuron by comparing the frequency of oscillation with a reference frequency. In contrast, thanks to the advances in power detector (PD) circuits, signals with few nano-Watt output power are detectable within few nano-seconds and with micro-Watts power consumption (Li et al., 2010; Qayyum and Negra, 2017). Hence, the output power of oscillation is used to detect the oscillating STNO.

The schematic view of the sensing approach is shown in Figure 4C. The current of memristor array passing through the STNO leads to its resistance oscillation. As a result, a weak signal with milli-Volt amplitude oscillating at GHz frequency will appear across the STNO. This weak AC signal, first, will be amplified by a low noise amplifier (LNA). Then, the output signal of LNA will be converted to a DC voltage by the PD. The output voltage of the PD will be compared with a threshold voltage by the comparator. In case, I_*Mem.*_ passing through the STNO will be high enough, the DC output voltage of the PD becomes higher than the threshould voltage. Hence, the output voltage of the comparator switches from “0” to “1” and it will be considered as neuron firing.

## Results

In order to evaluate the power efficiency of the LAO-NCS, first, the effect of elevating the temperature on the STNO characteristics is measured. Then, based on the measured results, a behavioral model of the STNO is extracted. For TaO_*x*_ memristor, a behavioral model for the temperature dependency of its resistance is extracted based on the data of Graves et al. (2017). Finally, both models are used to measure the power consumption of LAO-NCS in MATLAB simulator. The CMOS interface circuit is simulated and validated by HSPICE simulator in 65 nm CMOS technology. The thermal interaction between the laser pulses and the STNO is simulated in COMSOL simulator.

### Experimental Measurement

**FIGURE 5:**
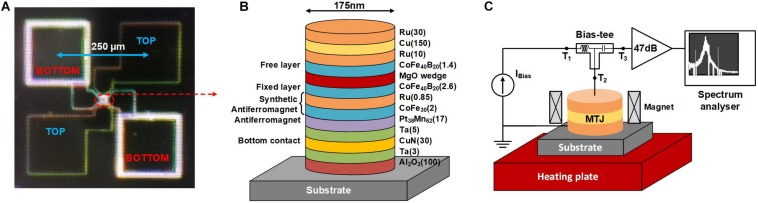
**(A)** The microscopic image and **(B)** schematic view of the MTJ stack as STNO. **(C)** Schematic view of the experimental setup used for characterization of the STNO at different temperatures.

In order to explore the effect of rising temperature on the STNO characteristics, we used the STNO stack structure of Substrate/(100) Al_2_O_3_/(3) Ta/(30) CuN/(5) Ta/(17) Pt_38_Mn_62_/(2) CoFe_30_/(0.85) Ru/(2.6) CoFe_40_B_20_/MgO wedge/(1.4) CoFe_40_B_20_/(10) Ru/(150) Cu/(30) Ru (thicknesses in nm). The CoFeB FL has in-plane magnetization. The stack has the circular shape with diameter of 175 nm. The microscopic image of the STNO sample and the schematic view of the deposited layer stack are shown in Figures 5A,B.

**FIGURE 6:**
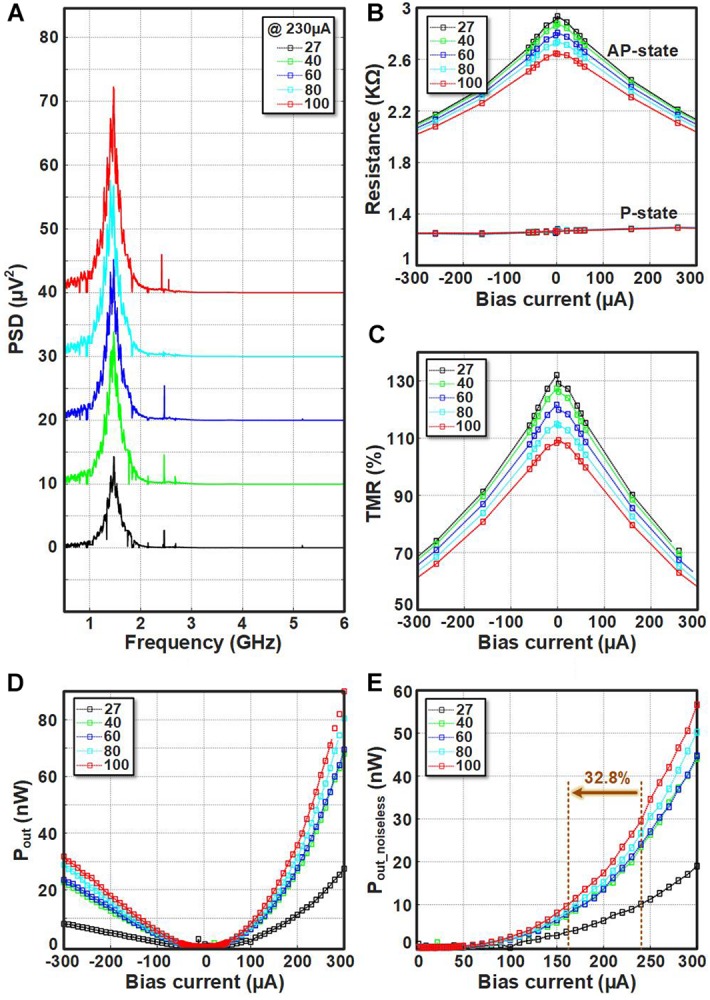
**(A)** The PSD measured at different temperature from 27°C to 100°C for 230 μA bias current, the curves are offset by 10 μV^2^ along the vertical axis for clarity. **(B)** The AP and P states resistance, **(C)** the TMR ratio, **(D)** the integrated matched output power (P_*out*_), and **(E)** the noiseless output power of STNO sample.

To evaluate the output power of the STNO at different temperatures, the experimental setup of Figure 5C is utilized. The bias current is injected to the STNO through T_1_ and T_2_ terminals of the bias-tee. In case, the bias current will be high enough, it will lead to the oscillation of the STNO resistance. This resistance oscillation will provide a micro-volt oscillation at T_3_ terminal of the bias-tee. Finally, the micro-volt oscillation of the STNO is amplified by an amplifier (47 dB) and will be injected to a spectrum analyzer in order to measure the oscillation characteristics of the STNO. The heating plate is used to set the temperature of the STNO at different temperatures above the room temperature. Figure 6A shows the PSD measured at different temperature from 27°C to 100°C for 230 μA bias current (the curves are offset by 10 μV^2^ along the vertical axis for clarity). Note that the impedance mismatch in the acquired spectrum needs to be considered. The input impedance of the amplifier is 50Ω. Hence, considering the resistance mismatch between the amplifier and the STNO, the measured output power is only a fraction of actual emitted power of the STNO. In order to eliminate the effect of impedance mismatch, the integrated matched output power (P_*out*_) of each device is calculated as follows (Costa et al., 2017):

(5) $$P_{o\,u\,t}=P_{m\,e\,a\,s\,u\,r\,e\,d}\,\left(\frac{{\left(R_{S\,T\,N\,O}+R_{A\,m\,p}\right)}^{2}}{4\,R_{S\,T\,N\,O}.R_{A\,m\,p}}\right)$$

where R_*STNO*_ and R_*Amp*_ are the resistance of the STNO and input resistance of the amplifier, respectively. P_*measured*_ is the measured output power based on the spectrum analyzer output. Figure 6B shows the measured STNO resistance in P- and AP-state at different temperatures from 27°C to 100°C. The AP-state resistance is decreased with increasing the temperature and the P-state resistance is almost constant as predicted by equation (3), and shown experimentally before (Teixeira et al., 2010; Takeuchi et al., 2015; Hu et al., 2016). As a result, the TMR ratio decreases by increasing the temperature (Figure 6C) that shows the typical behavior of MTJs as a function of the bias current. The matched output power (P_*out*_) of the STNO versus the bias current at different temperatures from 27°C to 100°C is shown in Figure 6D. By applying sufficient positive bias current, the oscillation will start, and by further increasing the bias current, the amplitude of the oscillation increases, which leads to a higher output power. Although the decrease in TMR with the bias current and temperature give an adverse result, the total power increases due to the fact that the input power increase dominates. It should be noted that applying a negative bias current will not cause oscillation, but increases the noise power, which leads to a higher P_*out*_. In order to eliminate the effect of noise on P_*out*_, the output power of the negative bias currents are deducted from the output power of the positive bias currents as shown in spin Hall nano-oscillators (SHNOs) (Tarequzzaman et al., 2019). As a result, the minimum bias current needed to detect the STNO oscillation of the fired neuron by the sensing circuit will decrease. This decreases the total energy consumption of the LAO-NCS as will be discussed in section “Hand-Written Digit Recognition Application”.

### Laser-Spin Torque Nano-Oscillators Interaction

The laser-STNO heat transfer is simulated in the COMSOL multiphysics simulator for the STNO stack (Böhnert et al., 2017). The shape, material and sizing of each layer is exactly similar to the STNO stack used in the experimental measurements. The laser beam is illuminated on the top electrode of the STNO stack to heat up the overall temperature of it. Hence, the top electrode should absorb the maximum laser energy in order to achieve the maximum efficiency. The bottom electrode is made of CuN with a thickness of 30 nm, while the top electrode is made of AlSiCu. Hence, a nanosecond laser with 355 nm wavelength is used to decrease the transmissivity of electrode. The optical transmittance and reflectance of the electrode are around 0.13 and 0.25, respectively (Maruyama and Morishita, 1998). Note that, the transmitted laser will be absorbed by lower layers in the STNO stack and increases its overall temperature. Hence, the energy loss is just related to the reflected laser. This is considered for calculating the total energy consumption described in the next sections. This energy loss can be reduced by engineering the material and surface of the top electrode.

**FIGURE 7:**
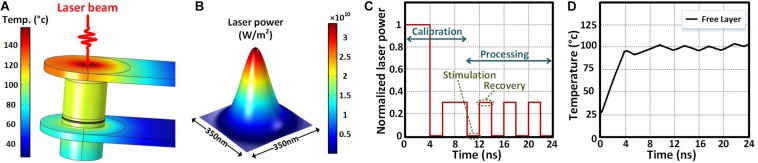
**(A)** The temperature distribution in MTJ stack at the end of calibration phase. **(B)** The laser power distribution. **(C)** The laser power pulse normalized to maximum laser power versus time. **(D)** The maximum and minimum temperature of MTJ stack and the FL temperature versus time.

Figure 7A shows the temperature distribution in the STNO stack at the end of calibration phase, which shows a uniform temperature distribution in all parts of the STNO. Figure 7B shows the laser power distribution. The power and the diameter of the laser beam are 71 μW and 350 nm, respectively. Figure 6C shows the normalized laser power in each laser radiation during the calibration and processing phases. The first two consecutive laser pulses do the calibration phase. The first laser pulse is illuminated for 4 ns with 100% power (71 μW) to heat the MTJ stack above 100°C. Then, the laser beam is cut off for 2 ns. The second laser pulse is applied for 4 ns with 30% power (21.3 μW) in order to stabilize the STNO temperature.

As mentioned before, the processing phase includes the stimulation and recovery steps. In the stimulation step, the inputs will be applied to the NCS and their corresponding response will be calculated by the NCS. The laser is cut off during the stimulation step. Then, the recovery step will be started and the input corresponding to the fired neuron will be activated in the post-synaptic neuronal layer. Moreover, the laser will be illuminated on the STNO with 30% power for 2 ns during the recovery step in order to compensate the heat-loss during the stimulation step. During the recovery step, the NCS inputs are disconnected and the power consumption of the STNOs and the memristor crossbar array is almost zero. This will continue repeatedly to keep the STNO temperature around 100°C. Figure 7D shows the temperature of the FL in the STNO stack, which is almost stabilized around 100°C (∼±5°C). It should be noted that the low temperature variations across the MgO barrier at the STNO stack prevents the reliability issues.

### Power Consumption of Spin Torque Nano-Oscillators and Memristor

**FIGURE 8:**
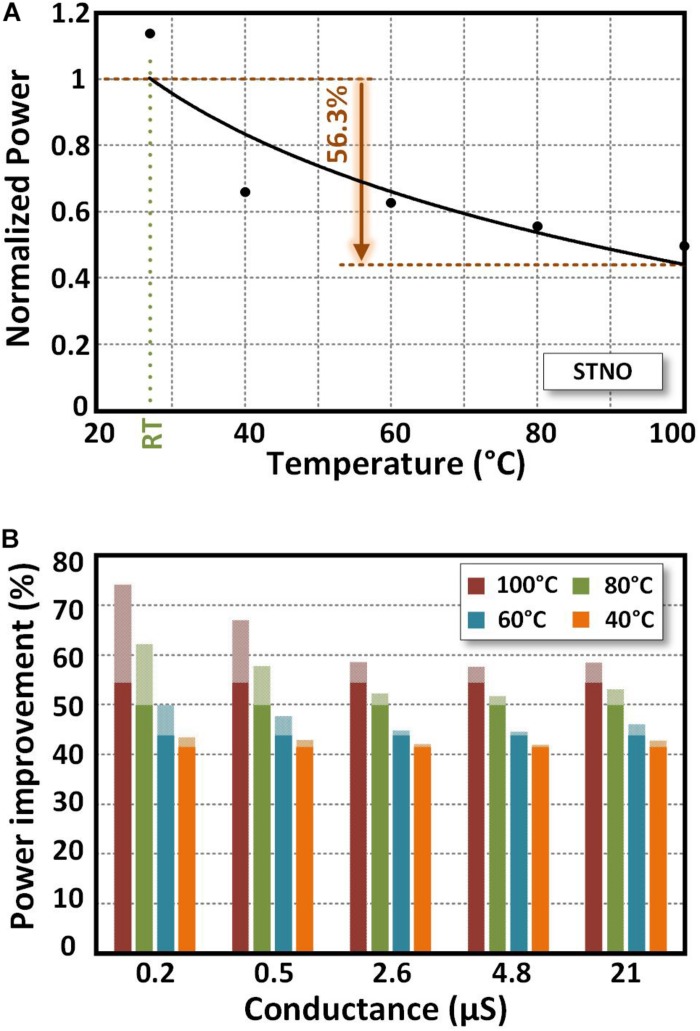
**(A)** The power consumption of an STNO vs. temperature. Power consumption is normalized to its room temperature value. **(B)** The power consumption improvement of memristor with different initial values at different temperature from 40°C to 100°C compared with room temperature (27°C). The solid part is related to memristor current reduction due to STNO current reduction. The dotted part is related to resistance reduction of memristor at elevated temperatures. In order to calculate the power consumption, the STNO and memristor current is supposed to be the minimum current required for starting oscillation (162 μA @ 100°C and 241 μA @ 27°C).

In this section, the effect of elevating the temperature on power consumption of the STNO and memristor is explored. Figure 8A shows the power consumption of an STNO at different temperatures. In order to calculate the STNO power consumption, the STNO current is supposed to be the minimum current required for starting the oscillation with a detectable output power, ranging from 241 μA at 27°C to 162 μA at 100°C (Figure 6E). The power consumption of STNO at the recovery step is zero. Hence, the calculated power consumption for the STNO is related to the stimulation step. The power consumption of the STNO decreases by 56.3% (127 μW @ 27°C to 55.5 μW @ 100°C) while increasing the temperature to 100°C. This is due to the fact that heating up the STNO reduces its magnetization saturation and effective anisotropy field that tends to keep the magnetization direction of the FL aligned with the easy axis. Note that in real applications, the STNO current depends on the input voltages of the memristor array and the initial resistance of the memristors. Hence, the real power consumption improvement is application-dependent.

In order to calculate the power consumption of TaO_*x*_ memristors at elevated temperatures, the conductance of the memristors and the current passing through them should be measured at different temperatures. However, the amount of conductance increase depends not only on the temperature, but also is a function of the initial resistance of the memristor (weights) and the applied voltage (inputs). Figure 8B shows the power consumption improvement of the TaO_*x*_ memristors with different initial conductance from 0.2 μS to 21 μS at different temperatures from 40°C to 100°C compared with the room temperature (27°C). The solid part is related to memristor current reduction due to the STNO current reduction and the dotted part is related to the resistance reduction of the memristor at elevated temperatures. The conductance range used in Figure 8B is aligned with the experimental data of Graves et al. (2017) that is used for benchmarking. Considering the fact that the STNO current will be passed through the memristors, the memristor current is considered equal with the STNO current at different temperatures when calculating the memristor power consumption.

As illustrated in Figure 8B, by increasing the memristor temperature to 100°C, the power consumption reduction of 59% for memristors with initial conductance values equal or lower than 2.6 μS is expected. However, for memristors with higher initial conductance values, the conductance increase rate due to the increased temperature is higher which leads to a larger power reduction. Note that most of the power consumption improvement of the memristors is due to the lower current passing through them (e.g., 4.8 μS at 100°C: 54.4% power improvement due to lower current versus 3% power improvement due to resistance reduction), especially at conductance values equal or lower than 2.6 μS. On the other hand, heating up the memristor array requires a laser pulse with higher output power, which reduces the power efficiency of the proposed LAO-NCS. Hence, in the LAO-NCS, the laser is used to heat up the STNOs only and the temperature of memristor array leaved unchanged.

### Hand-Written Digit Recognition Application

**FIGURE 9:**
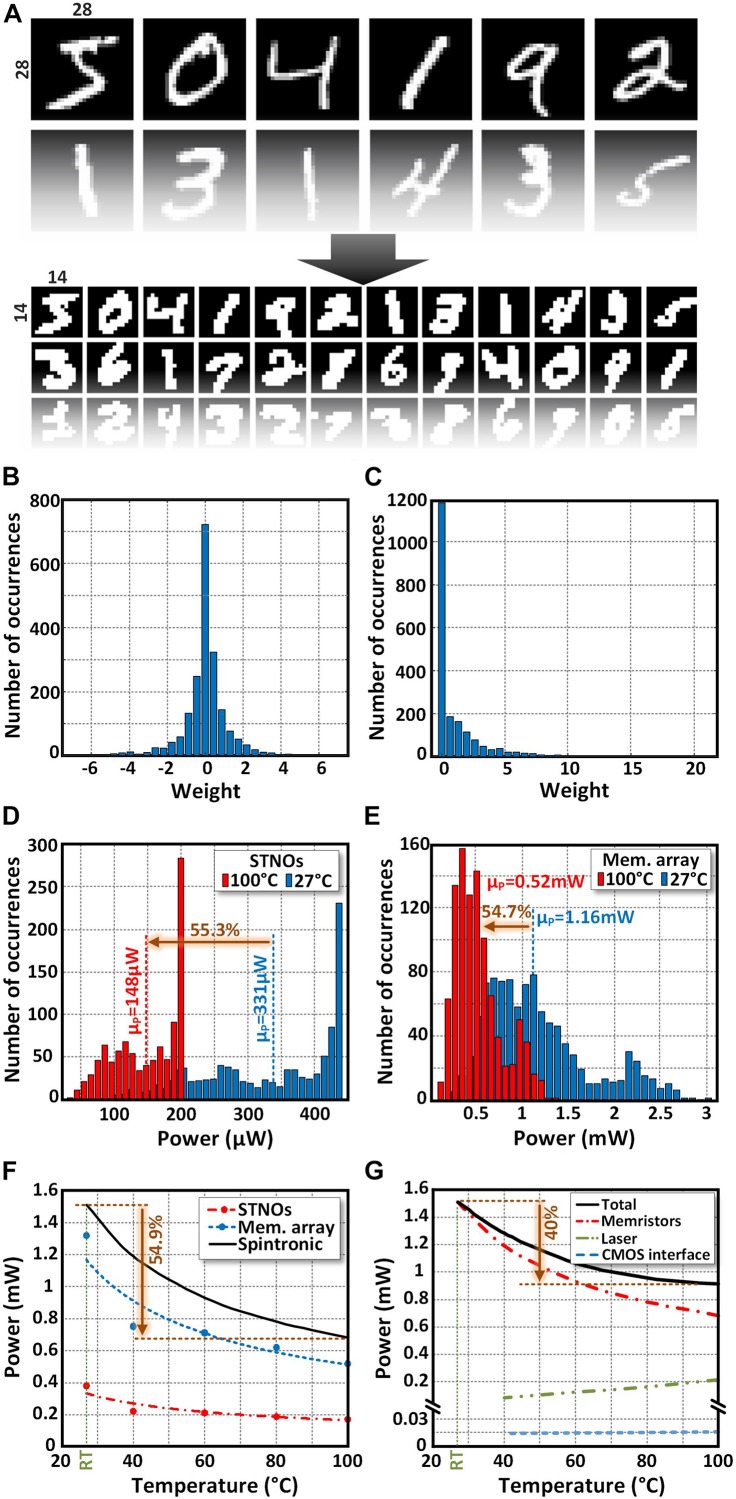
**(A)** The size of pictures in MNIST database is reduced to 14 × 14 and used to train the 196 × 10 NCS. **(B)** The original weights after training. **(C)** Weights after setting the negative ones to zero and multiplying the positive ones by 3. The power consumption distribution of **(D)** STNOs and **(E)** memristor array in 196 × 10 NCS for 1000 test images at 27°C and 100°C, respectively. The average power consumption of **(F)** STNOs, memristor array, **(G)** laser, CMOS interface circuit, and the whole NCS at different temperatures from 27°C to 100°C.

Considering the fact that independent studies of spintronic elements cannot accurately reflect the performance of the whole NCS, the effectiveness of the proposed LAO-NCS is evaluated by the hand-written digit recognition application. To do that, a 196 × 10 NCS is designed to recognize the handwritten digits in MATLAB. Then, the MNIST handwritten digits database (LeCun et al., 1998) is used to train the NCS and the weights are extracted. The network is trained by 1000 training images using the Scaled Conjugate Gradient (SCG) method for a fully connected feedforward neural network. The size of the images is reduced to 14 × 14 (Figure 9A). Considering the facts that the negative weights cannot be implemented by the memristors, the negative weights are considered as zero (Figures 9B,C). This will reduce the accuracy of system (89% → 54%). In order to compensate the accuracy reduction partially, the positive weights are multiplied by three. This will increase the accuracy from 54% to 71.3%. In the next step, the weights are mapped to the resistance of memristors in the array (the zero weights are considered as open circuit). In order to model the effect of temperature increase on the power consumption of the STNOs, equations are fitted to the experimental results of section “Interaction between Laser and the STNOs” (Figure 6). Then, the fitted equations are used in MATLAB to model the power consumption of the LAO-NCS at different temperatures from 27°C to 100°C. Considering the fact that the laser just illuminated on the STNOs, the temperature of the memristor array is assumed to be lower than 350K (the memristor resistance is constant with respect to its temperature). However, in case, the temperature of the memristor array increases due to heat propagation from the STNOs, the resistance of the memristors slightly reduces that improves the power efficiency of the LAO-NCS. Finally, 1000 test images have been applied to the modeled NCS in MATLAB and the power consumption reduction of the STNOs (Figure 9D) and the memristor array (Figure 9E) is calculated for each test image. The accuracy of the system is 71.3%. This is due to the fact that the negative weights cannot be implemented using memristors. The accuracy can be improved by adding a hidden layer to the system or adding the number of neurons in each layer, which comes with a higher complexity in hardware implementation. However, the main focus of this manuscript is on investigating the effect of raising temperature on the power consumption of the STNO-based NCS.

As illustrated in Figures 9D,E, increasing the temperature from 27°C to 100 has reduced the average power consumption of the memristor array and the STNOs by 54.7% and 55.3%, respectively. Hence, the total power consumption of the spintronic layer is reduced by 54.9% as shown in Figure 9F. This is due to the smaller resistance of the STNOs and the lower bias current passing through the memristors and the STNOs at elevated temperatures. The average power consumption improvement of the STNOs (54.9% in for a single STNO (56.3% in Figure 8A) in previous section. This is due to the fact that the calculated power consumption is the average of power consumption of all STNOs (the oscillating one and the non-oscillating STNOs).

The total power consumption of the LAO-NCS includes the power consumption of the spintronic layer (the memristors and the STNOs), the CMOS interfacing circuit, the CMOS sensing circuit and the laser. However, it should be noted that the sensing circuit is common between the LAO-NCS and typical STNO-based NCS. Hence, it has similar effect on the total power consumption of both circuit. Due to the fact that the calibration phase is done just one time at the beginning of the NCS operation, its power consumption’s contribution to the total power consumption of the LAO-NCS is negligible. Hence, the power consumption of the LAO-NCS is calculated only for the processing phase. The power consumption of the CMOS interfacing circuit and the laser are shown in Figure 9G at different temperatures. The power consumption of the CMOS interfacing circuit can be further decreased using low voltage circuit techniques. However, due to its very low power consumption (∼15μW), its effect on the total power consumption is negligible. To achieve a higher temperature, a higher laser power is required. Note that, in stimulation step, the laser is turned off and it has no power consumption. In recovery step, the laser is illuminated for 2 ns with 213 μW power to keep the temperature of the STNOs around 100°C. Considering the fact that the power consumption improvement in the spintronic layer is significantly higher than the power consumption of the laser and the CMOS interfacing circuit, the total power consumption of the LAO-NCS decreases by 40% at 100°C compared with the room temperature.

The power consumption of the CMOS sensing circuit can be estimated as the total power consumption of LNA, PD, and comparator. The power consumption of LNA at required frequency range (1.3–1.5 GHz, Figure 4A) can be estimated around 160 μW (Parvizi et al., 2016). However, this is a wideband LNA and the power consumption of narrowband LNA can be lower (Kargaran et al., 2018). Moreover, PD circuits with power consumption lower than 100 μW are realized in the literatures (Li et al., 2010; Qayyum and Negra, 2017). Finally, the power consumption of the comparator (@500 MHz) can be estimated 200 μW (Khorami and Sharifkhani, 2018). All in all, the total power consumption of the CMOS sensing circuit is estimated to be lower than 400 μW for this specific application.

#### Technology Scaling Effect on LAO-NCS

Technology scaling will lead to a lower laser power consumption due to the smaller size of the STNOs. As a result, the power efficiency of the LAO-NCS is expected to improve further. As an example, for the STNO samples of Monteblanco et al. (2017), the STNO area is 60 nm × 70 nm = 4200 nm^2^. Comparing with the STNO samples used in this manuscript with 24052 nm^2^, the STNO samples of Monteblanco et al. (2017) need a laser pulse with ∼ 38 μW output power in order to increase their temperature up to 100°C. Decreasing the laser power from 213 μW to 38 μW reduces the total power consumption of the NCS at 100°C from 911 μW to 736 μW in Figure 9G. As a result, the power consumption improvement of the LAO-NCS will be increased from 40% to 51.3%.

#### Energy Consumption

Elevating the temperature reduces the switching time of the MTJs (Farkhani et al., 2019b). Since the oscillation mechanism of STNOs is similar with switching, similar trend is expected for the delay before starting the oscillation. Hence, at 100°C, 77% delay reduction can be expected (Farkhani et al., 2019b). The energy consumption can be calculated from multiplication of power consumption and delay. Hence, considering 77% delay reduction and 40% power consumption reduction, 86% lower energy consumption of the LAO-NCS at 100°C can be expected compared with a typical STNO-based NCS at room temperature.

#### Comparison With CMOS-Based NCS

Considering the fact that there is no fully-implemented and integrated spintronic-based NCS, it is hard to perform an accurate comparison between spintronic-based NCS and the CMOs-based NCS. Hence, it is tried to give a general perspective. Synaptic memristors (130 × 10^15^) shows 10–100 times better performance (operation/sec/Watt/cm^2^) over CMOS-based synapses (∼2 × 10^15^) (Mandal et al., 2014). Furthermore, 2–3 orders of magnitude improvement by MTJ neurons and their sensing circuit (1.2 × 10^8^) is achieved over fully-CMOS implementations (2.3 × 10^5^) (Mizrahi et al., 2018). Neurons and their sensing circuits contribute the most to the overall performance. Therefore, 2–3 orders of magnitude performance improvement is expected using MTJ-Memristor NCSs compared with the CMOS-based NCSs. The use of nano-oscillators specified for NCSs, one order of magnitude improvement in performance compared to the use of MTJ neuron, where full switching is used [critical current density: ∼10^6^ A/cm^2^ (Costa et al., 2017) vs. ∼10^7^ A/cm^2^ (Fukami et al., 2016)], is expected. Finally, thermally assisting STNOs using laser can improve the power consumption by 40%. However, the spin-based devices are suffering from high process variation and relatively high cost compared with their CMOS counterpart.

### Comparison With the Other Heating Methods

Thermally assisted MTJ switching in STT-RAMs is widely used to decrease the bias current (Walter et al., 2011; Prejbeanu et al., 2013; Bender and Tserkovnyak, 2016; Dai et al., 2017; Safranski et al., 2017). Heating up the MTJ is used to improve the FL switching in two different ways including creating a temperature gradient between FL and PL, called Seebeck effect (Walter et al., 2011; Bender and Tserkovnyak, 2016; Safranski et al., 2017) and heating the MTJ above FL blocking temperature (T_*B*_) to reduce its switching current, called Thermally Assisted Switching – TAS (Prejbeanu et al., 2013; Bandiera and Dieny, 2016; Dai et al., 2017). In the first method, using a temperature gradient across the MTJ, a pure spin current will be injected to FL. This pure spin current acts as an anti-damping thermal spin torque (also called spin Seebeck torque) and decreases the bias current (Safranski et al., 2017). Note that in NCS application, heating up the MTJs should not lead to their switching, but heating should ease the switching. This means that the switching should happen by the current flowing from crossbar array with the help of heating. Hence, the Seebeck effect, in original form, cannot be used in NCSs. Moreover, considering the fact that temperature gradient is the source of Seebeck effect, specific time should be allocated for MTJ cooling before starting the next stimulation step that lowers the general speed of the NCS.

In TAS, a modified type of MTJ is needed, where the FL consists of a ferromagnetic layer pinned with a low T_*B*_ antiferromagnet (AF), such as FeMn (90–160°C) or IrMn (120–260°C). The PL is a SyF pinned with a high T_*B*_ antiferromagnet, such as PtMn (350°C) (Bandiera and Dieny, 2016). In standby mode, the FL presents a very high thermal stability, because it is pinned by the low T_*B*_ antiferromagnet. Then, during stimulation phase, the stack heats up in order to ease the FL switching (Prejbeanu et al., 2013). In TAS, Joule heating is used to heat the MTJ junction above the blocking temperature of antiferromagnetic layer by passing an extra current through it. Then, a magnetic field or a spin polarized current switches the FL magnetization. Finally, while keeping the magnetic field or spin polarized current, the MTJ stack is cooled down.

#### Energy Consumption

In TAS, in order to heat the FL layer by 200°C, a current density of 2–4 × 10^6^ A.cm^–2^ with a bias voltage of 1.1V is needed for different materials (Prejbeanu et al.; 2013). Hence, the power consumption of the TAS is estimated as 0.53 mW to 1.06 mW for the MTJ stack with a cross section area similar to the MTJ stack used in our simulations [π × (87.5 nm)^2^ = 24.052 × 10^–15^m^2^]. The power consumption of LAO for a 200°C temperature increase of the STNO is estimated as 0.4 mW, which shows 1.3X-2.6X lower power consumption compared with the TAS. In addition, the use of LAS has the following advantages over TAS:

(1) The TAS, in its original form, cannot be used in an NCS application because heating all the MTJs above T_*B*_ and passing current through them leads to FL switching in all MTJs.
(2) There is no need for an antiferromagnetic layer close to the FL and the proposed laser assisted method can be applied to typical MTJs.
(3) No heating current line is required, which improves the density.
(4) In contrast with the TAS, which needs a bipolar select transistor in order to inject two bipolar current into the MTJ, LAS can be used with CMOS select transistor.
(5) In TAS, the minimum heating time is limited to 500 ps (Bandiera and Dieny, 2016) due to the fact that the MTJ voltage should not exceed the MTJ breakdown voltage. However, the heating of the ferromagnetic material above Curie temperature by a femto-second laser pulse has been shown experimentally (Walowski, 2012).

In terms of complexity, considering the extra layer (photonic layer) needed in the implementation of the LAO, LAO comes with a higher complexity compared to the Joule heating approach.

## Conclusion

To reduce the power consumption of future STNO-based NCSs, a microwatt-nanosecond laser pulse is utilized for the first time to ease the magnetic oscillation of the STNO through heating. The power consumption of the spintronic layer and the total power consumption of the proposed LAO-NCS are improved by 54.9% and 40% at *T* = 100°C compared with operation at the room temperature. Moreover, 86% lower energy consumption can be expected for the LAO-NCA at 100°C compared with a typical NCS at the room temperature. It should be noted that scaling the technology and increasing the temperature above 100°C leads to further improvement of the power consumption.

## Data Availability Statement

The raw data supporting the conclusions of this article will be made available by the authors, without undue reservation, to any qualified researcher.

## Author Contributions

HF and FM designed and performed the research, and wrote the manuscript together with TB and JM. JC, MT, and RF designed and fabricated the STNO samples for testing and characterization that was done by HF, TB, MT, AJ, and RF.

## Conflict of Interest

The authors declare that the research was conducted in the absence of any commercial or financial relationships that could be construed as a potential conflict of interest.
